# Urbanization affects neophilia and risk-taking at bird-feeders

**DOI:** 10.1038/srep28575

**Published:** 2016-06-27

**Authors:** Piotr Tryjanowski, Anders Pape Møller, Federico Morelli, Waldemar Biaduń, Tomasz Brauze, Michał Ciach, Paweł Czechowski, Stanisław Czyż, Beata Dulisz, Artur Goławski, Tomasz Hetmański, Piotr Indykiewicz, Cezary Mitrus, Łukasz Myczko, Jacek J. Nowakowski, Michał Polakowski, Viktoria Takacs, Dariusz Wysocki, Piotr Zduniak

**Affiliations:** 1 Institute of Zoology, Poznań University of Life Sciences, Wojska Polskiego 71C, PL-60-625 Poznań, Poland; 2 Ecologie Systématique Evolution, Université Paris-Sud, CNRS, AgroParisTech, Université Paris-Saclay, F-91405 Orsay Cedex, France; 3 Czech University of Life Sciences Prague, Faculty of Environmental Sciences, Department of Applied Geoinformatics and Spatial Planning, Kamýcká 129, 165 00 Prague 6, Czech Republic; 4 Faculty of Biological Sciences, University of Zielona Góra, Institute of Biotechnology and Environment Protection, Prof. Szafran St. 1, PL 65-516 Zielona Góra, Poland; 5 High School of Civil Sciences, Zamojska 47, PL-20-1012 Lublin, Poland; 6 Department of Vertebrate Zoology, Faculty of Biology and Environment Protection, Nicolaus Copernicus University, Lwowska 1, PL-87–100 Toruń, Poland; 7 Department of Forest Biodiversity, University of Agriculture, Al. 29-Listopada 46, PL-31-425 Kraków, Poland; 8 Institute for Administration and Tourism, State Higher Vocational School in Sulechów, Armii Krajowej Str. 51, PL-66-100 Sulechów, Poland; 9 Upper Silesian Ornithological Society, pl. Jana III Sobieskiego 2, PL-41-902 Bytom, Poland; 10 Department of Ecology & Environmental Protection, University of Warmia and Mazury in Olsztyn, Plac Łódzki 3, PL-10–727 Olsztyn, Poland; 11 Department of Zoology, Siedlce University of Natural Sciences and Humanities, Prusa 12, PL-08–110 Siedlce, Poland; 12 Department of Zoology, Pomeranian University, Arciszewskiego 22b, PL-76-200 Słupsk, Poland; 13 Department of Zoology and Landscaping, University of Technology and Life Sciences, Ks. A. Kordeckiego 20, PL-85-225 Bydgoszcz, Poland; 14 Department of Zoology, University of Rzeszów, Zelwerowicza 4, PL-35-601 Rzeszów, Poland; 15 Department of Environmental Protection and Management, Bialystok University of Technology, Wiejska 45a, PL-15-351 Białystok, Poland; 16 Department of Vertebrate Zoology and Anthropology, University of Szczecin, Wąska 13, PL-71-412 Szczecin, Poland; 17 Department of Avian Biology & Ecology, Faculty of Biology, Adam Mickiewicz University, Umultowska 89, PL-61-614 Poznań, Poland

## Abstract

Urban environments cover vast areas with a high density of humans and their dogs and cats causing problems for exploitation of new resources by wild animals. Such resources facilitate colonization by individuals with a high level of neophilia predicting that urban animals should show more neophilia than rural conspecifics. We provided bird-feeders across urban environments in 14 Polish cities and matched nearby rural habitats, testing whether the presence of a novel item (a brightly coloured green object made out of gum with a tuft of hair) differentially delayed arrival at feeders in rural compared to urban habitats. The presence of a novel object reduced the number of great tits *Parus major*, but also the total number of all species of birds although differentially so in urban compared to rural areas. That was the case independent of the potentially confounding effects of temperature, population density of birds, and the abundance of cats, dogs and pedestrians. The number of great tits and the total number of birds attending feeders increased in urban compared to rural areas independent of local population density of birds. This implies that urban birds have high levels of neophilia allowing them to readily exploit unpredictable resources in urban environments.

The extent of urban areas is increasing rapidly worldwide caused by the world’s human population growing at an unprecedented rate, and most people clustering around urban areas^1^. Cities around the globe have experienced unparalleled population growth with 10% of humans living in cities in 1900, 50% in 2000, and 70% are predicted to live in cities by 2050^2^. These effects of urbanization have dramatic consequences on all living beings at the level of cellular mechanisms, life history, demography, interspecific interactions, communities and biodiversity^2^,^3^.

However, urbanisation does not affect all individuals equally because different animals with different personalities vary in their ability to cope with urban environments. Individuals can respond to novel stimuli in at least one of three ways: with interest (neophilia), fear (neophobia), or indifference^4^. For example, neophobic individuals show reduced ability to cope with novel foraging tasks^5^,^6^,^7^. Generally urban environments hold superabundant food sources^2^,^3^, and neophilia combined with exploration may facilitate exploitation of such novel food^4^,^8^,^9^. Exploitation of novel food may especially occur when neophilic individuals invade novel habitats^10^. For example, urban individuals of common mynas *Acridotheres tristis* are more neophilic than suburban conspecifics^11^, and flocks of house sparrows *Passer domesticus* from urban environments solve tasks more quickly than flocks of rural conspecifics, indicating advantages of neophilia^12^. Repeated exposure to novel objects, like bird-feeders, may reduce the degree of neophobia^13^, and eventually individuals living in urban environments may show decreased levels of neophobia as a result of repeated exposure to novelty^9^,^14^,^15^. Such attraction to novelty defined as neophilia has been described repeatedly^16^. Other potential mechanisms affecting the distribution of specific phenotypes in urban areas include differential dispersal of individuals that can cope with humans and evolutionary adaptation to human proximity^17^,^18^,^19^,^20^.

Bird-feeders are ubiquitous across the globe with vast quantities of food and other resources for animals occurring in urban environments. Such food provisioning increases the fat load of birds^21^. Therefore, feeders play a significant role as determinants of important life history traits such as survivorship, phenology and fecundity^22^. Recent studies have shown that population density of urban birds has more than doubled compared to that of rural birds^23^,^24^. Bird-feeders are particularly commonly exploited in urban habitats, especially in places where more feeders are available, although this effect is modified by the presence of predators such as cats^25^.

Here we assessed factors that contribute to coping with novelty by individual birds when we displayed a brightly coloured unfamiliar but neutral object, which does not resemble any predator, competitor or conspecific, compared to a control treatment^26^. As a novel object we used a typical children’s toy (oval shape, 8 cm in length and 4 cm in width) that is brightly green and made out of gum with a tuft of hair. The object was not previously shown to the birds, and we never observed anything even vaguely similar in the field making it highly unlikely that local birds responded to this object as anything but a novel object. The object was fixed on top of a standard bird-feeder at the start of an observation session, whereas in the control group the feeder was missing the novel object.

The aims of this study were to experimentally test for an effect of neophilia on exploitation of a novel food resource as reflected by a novel bird-feeder with a novel and brightly coloured object attached. We predicted that the level of neophilia would be lower in rural habitats, based on the assumption that the local bird community is less experienced with novel food sources. In contrast, we predicted a greater rate of recruitment to such a novel resource in urban habitats, even for a given level of population density, than in rural habitats, simply because urban birds are often innovative in their exploitation of the environment^27^. Thus we expected an interaction between urban vs. rural habitat and the effect of presence or absence of the brightly coloured object. That should even be the case when adjusting for the potential risk of predation due to cats, and the presence of dogs and humans, which may also be perceived as threats. We tested these predictions in an experiment during winter across 14 cities and nearby rural areas in Poland.

## Results

In total 1845 individuals belonging to 19 bird species were recorded at bird-feeders, but four species (great tit *Parus major*, blue tit *Cyanistes caeruleus*, greenfinch *Chloris chloris* and tree sparrow *Passer montanus*) constituted almost 90% of the entire bird community (Table 1).

Because the great tit was the most common species in the study with a total sample size allowing a separate analysis for this species, we also provided results for great tit, which is known as a species frequently using bird-feeders^9^,^20^,^24^,^25^.

### Great tits and novel objects

All predictor variables except whether trials were classified as early or late and temperature explained significant variation in the abundance of great tits (Table 2). Presence of the novel object strongly reduced the number of great tits compared to when the novel object was absent (Table 2). More great tits visited feeders in urban habitats and at high population density of great tits and other bird species near the feeder (Table 2). There were more great tits at feeders at high density of cats, but fewer tits at high density of dogs (Table 2). Finally, there were more great tits in the presence of more pedestrians (Table 2). There was an interaction between presence of the novel object and habitat: There were more great tits in rural habitats in the absence than in the presence of the object, while in urban habitats there were more great tits in the presence than in the absence of the object (Table 2).

### Overall abundance of bird species and presence of the novel object

**Figure 1:**
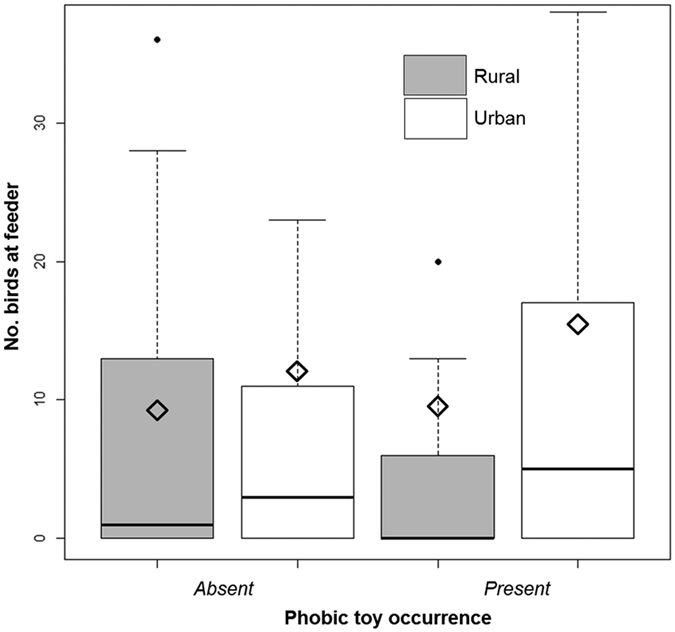
Number of individual birds at feeders in relation to urbanisation and presence of a novel object. Box plots show medians, quartiles, 5- and 95-percentiles and extreme values.

**Figure 2:**
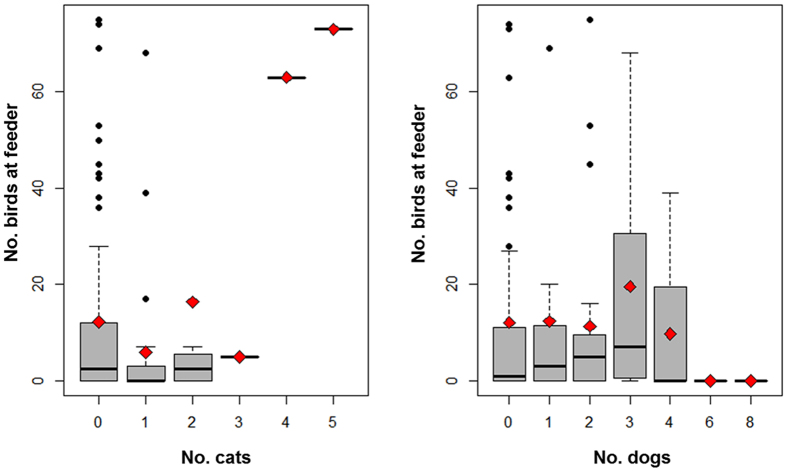
Number of individual birds at feeders in relation to number of cats and dogs. Box plots show medians (black horizontal bars), mean (empty rhombus), quartiles, 5- and 95-percentiles and extreme values.

The results were generally similar to those obtained for great tits, but with slight differences mainly in effect size of impact of particular explanatory variables if all species of birds visiting the bird-feeders were included. There was an intermediate effect of the novel object on the abundance of birds with fewer birds present in the presence of the object (Table 3; Fig. 1). There was no significant effect of cold weather on the number of birds (Table 3). There was an intermediate effect size for habitat with more birds being present in cities (Table 3). Moreover, when there were more birds present in the neighbourhood, this increased the number of birds at the feeders with a large effect size (Table 3). Furthermore, the presence of more cats increased the number of birds at feeders with an intermediate effect size (Table 3; Fig. 2). There was a strong effect for dogs negatively affecting the abundance of birds at feeders, whereas the effect size for pedestrians was positive and weak (Table 3; Fig. 2). Finally, there was an intermediate effect size for the interaction between habitat and presence of the object (Table 3). While the presence of the toy increased the abundance of birds in urban habitats, there was an opposite effect in rural sites (Table 3).

## Discussion

Urban environments have superabundant, but often unpredictably distributed food, mainly of anthropogenic origin putting a premium on individual birds that are able to rapidly locate such resources^24^,^28^. Neophilia has been hypothesised to be a major characteristic of such superior exploiters, although the evidence and the generality (in terms of species and habitats) of this effect remains to be determined^4^,^11^,^27^. Indeed, among urban individuals food driven object exploration is faster^11^, being more neophilic than suburban conspecifics^29^. Urban habitats were disproportionately exploited by birds that either disregarded or were even attracted to a novel stimulus (a brightly green novel object with a tuft of hair). In contrast, rural birds remained at a distance from the feeders and the novel object. This provides experimental evidence for neophilia in urban birds promoting exploitation of resources, although the experiments do not allow distinction of the origin of the behavioural differences (experience dependent vs. independent). One the most probable explanations is that urbanisation of birds results in differential recruitment of individuals with higher levels of neophilia to urban habitats. Once individuals with higher levels of neophilia have been attracted to urban habitats with its super-abundant food, they may enjoy differential viability and fecundity compared to individuals from rural populations^18^,^20^.

Recruitment in winter to novel bird-feeders by common bird species depended on the presence of a novel object and, importantly, the interaction between habitat and object. We interpreted this difference as an effect of exploration and neophobia^4^. Because so many birds, both species and individuals, responded differentially to the presence of a novel object in the urban, compared to the the rural habitats, we suggest that colonization of urban habitats is by individuals with high levels of exploratory behaviour and low levels of neophobia allowing for exploitation of such novel environments. We documented similar effects in great tits and in the urban bird community at large, although the effect size was larger for tits than for the entire community, because great tits explored bird-feeders faster than other birds^25^. The statistically significant and large interaction between habitat and presence of the novel object implies different patterns of feeder use in the presence and the absence of the novel object. There are dangers of novel food^29^ including exposure to the enemies living in the neighbourhood of such novel feeders^9^,^30^,^31^,^32^. Our experiments do not allow discrimination between effects of habituation, differential dispersal of phenotypes and micro-evolutionary change as determinants of these behavioural differences between paired rural and urban study plots^19^,^33^.

Birds are known to exploit feeders at low temperature, and when population density is high^24^,^34^, although our experiments only partially confirmed these well-known effects. Urbanisation is associated with an increase in the abundance of cats and dogs^24^,^35^, but a decrease in the abundance of raptors that keep a safe distance from humans^36^. We suggest that the different reactions of birds to dogs and cats is related to the behaviour of these two mammals. While cats are commonly free-ranging and wild, dogs mainly but not always walk with a collar in the company of human pedestrians, and have a tendency to disturb birds^37^. However, even humans may cause high levels of disturbance for animals in urban habitats thereby causing birds to incur high metabolic costs of displacement^36^,^38^. We hypothesise that cats and dogs are attracted to feeders^39^ thereby reducing exploitation of feeders by birds^25^. An aggregation of cats and dogs at feeders in urban habitats may force birds to trade exploitation of food against safety from predators as shown by patterns of flight initiation distances between rural and urban habitats^35^. At this stage, conclusions are difficult without experiments dedicated particularly to the presence of dogs and cats.

In conclusion, we have provided experimental evidence for differential recruitment of birds to novel bird-feeders showing that individuals with high levels of neophilia are more often recorded in urban, than in rural habitats. We hypothesise that this habitat difference interacting with the effect of low levels of avoidance of a novel stimulus (a brightly coloured toy) provides a mechanism for exploitation of urban environments.

## Methods

### Field study

**Figure 3:**
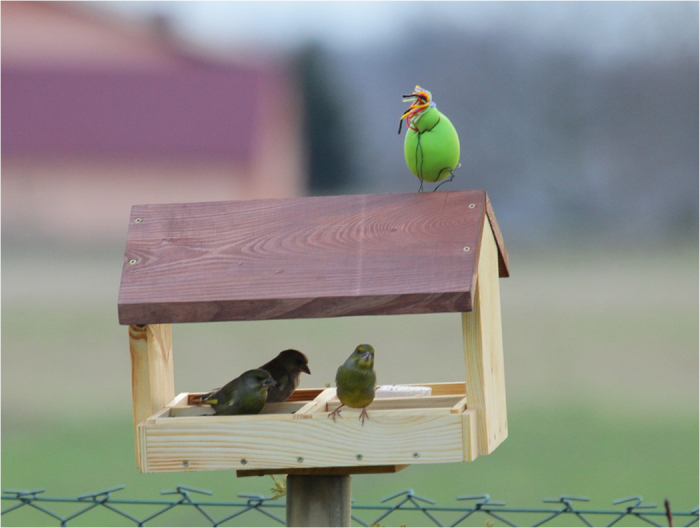
Bird-feeder with greenfinches *Chloris chloris* in the presence of the novel object.

Data were collected twice during December 22^nd^ 2013–February 18^th^ 2014 across Poland, within and outside 14 cities. In total 160 experimental trials (80 in cities and 80 in a nearby rural areas) were carried out 1–4 h after sunrise to reduce daily variation^39^ under favourable weather conditions (no precipitation, no strong wind). Identical, wooden bird-feeders (n = 80; 40 in urban and 40 in rural habitats) were used in all trials across the entire country, each feeder having the shape of a small house with a roof placed on top of a 1.20 m tine pole, to avoid mammals visiting especially during night. Observations and use of photo-traps did not reveal any mammals at the bird-feeder. The pole was dug into the ground (grass/soil) and provisioned in the late evening (after the end of daily activity of the birds, after 6 pm local time, to ensure that in all experiments feeders could be discover by birds only the following morning). Each bird-feeder contained at the bottom four different trays (their position was changed randomly for each trial) with four different kinds of food (containing carbohydrates and lipids): animal fat, sunflower seeds, millet seeds and dry fruits of rowanberry, respectively (each food substrate always covered 25% of the bottom area of the bird-table at the beginning of the experiment). The novel object (Fig. 3) was placed on the roof of a bird-feeder in a randomly chosen half of the experimental trials, with half in urban and the other half in rural areas.

We quantified the composition of the local wintering bird community at three census points at distances of 100 m from the feeder, located at virtual triangle tops with the bird-feeder in the middle. All birds seen or heard were recorded using a point count with 5-min observations at each point^4^, and once again to reduce daily variation in the number of birds monitored 1–4 h after sunrise^40^. This standard bird census method was tested during winter conditions in Poland for species presence and detectability^23^, and it provides reliable information on relative density of birds^41^. Data from the three point counts were summed and used to describe the composition of the winter bird community in the immediate neighbourhood of each feeder. During bird counts the number of cats and dogs within a distance of 100 m from feeders was also recorded, and additionally at the start of each experimental trial ambient temperature (in °C).

When a new experimental bird-feeder was provided at a specific site, it was observed for 30 min with a pair of binoculars from a parked car at a distance with good visibility. The observer noted the number of individuals of each species visiting each feeder.

Birds were not individually marked and therefore not individually identifiable, which might slightly affect the results. To avoid the problem of counting the same individuals twice, and thus to avoid pseudo-replication, experimental trials were performed at a distance of at least 1500 m between study sites.

### Statistics

We tested for spatial auto-correlation in behaviour and composition of bird communities across cities. We used Mantel tests^42^ for investigating spatial dependence of observations. The Mantel test evaluates the similarity between two matrices calculated as a geometric distance matrix, and when spatial autocorrelation exists, then the closer the plots are in geometric space, the more similar the pattern of values between matrices^43^. We used Monte Carlo permutations with 999 randomizations to test for statistical significance^44^. Sample sites were treated as statistically independent observations because the values of spatial autocorrelation were low and far from statistically significant (Mantel test: *r*_M_ = −0.021 *P* = 0.868).

Generalized linear mixed models were used in this study to predict the number of great tits and the total number of birds at each bird feeder. Both variables were described with a Poisson error distribution (that is adequate for count data) and the models were fitted by maximum likelihood (Laplace approximations) specifying a logit link function, using the package ‘lme4’^45^ in R^46^.

We made a separate analysis for great tits because it was by far the most common species recorded in the study (1318 individuals recorded among 1845 birds of all species across the 160 tests). The response variables were the number of great tits and the total number of individuals of all other bird species combined at the feeder. We combined all other species in an attempt to test for differences between tits and all other birds. As fixed effects we used as categorical explanatory variables presence or absence of the novel object and habitat (rural or urban) and as continuous explanatory variables temperature (predicting more birds at feeders when it was cold), density of great tits (or all bird species, predicting more birds at feeders when more birds were present in the neighbourhood), number of cats (predicting fewer birds when predatory cats were more abundant), number of dogs (predicting fewer birds when dogs were more abundant) and number of humans (predicting more birds when there were many humans around and hence more disturbance that keeps cats and dogs away). The presence of cats, dogs and human pedestrians was recorded directly in the field during observations of bird-feeders^25^. Finally, we included the interaction between habitat and presence of the novel object because we expected opposite results for the two habitats (urban or rural) in the presence and the absence of the novel object.

We assessed the strength of each predictor relying on effect sizes estimated in terms of Pearson’s product-moment correlation coefficients. We adopted the criteria listed by Cohen^47^ for small (*r* = 0.10, explaining 1% of the variance), intermediate (*r* = 0.3, explaining 9% of the variance) or large effect sizes (*r* = 0.5, explaining 25% of the variance).

## Additional Information

**How to cite this article**: Tryjanowski, P. *et al*. Urbanization affects neophilia and risk-taking at bird-feeders. *Sci. Rep.* **6**, 28575; doi: 10.1038/srep28575 (2016).

## Figures and Tables

**Table 1:** Bird species and number of individuals recorded at bird-feeders.

Species	No. observations	Frequency
*Parus major*	1330	72.09
*Cyanistes caeruleus*	116	6.29
*Chloris chloris*	111	6.02
*Passer montanus*	97	5.26
*Sitta europea*	36	1.95
*Passer domesticus*	28	1.52
*Coccothraustes coccothraustes*	28	1.52
*Turdus merula*	27	1.46
*Garrulus glandarius*	21	1.14
*Carduelis spinus*	18	0.98
*Pica pica*	9	0.49
*Streptopelia decaocto*	9	0.49
*Corvus monedula*	5	0.27
*Poecile palustris*	4	0.22
*Erithacus rubecula*	2	0.11
*Fringilla montifringilla*	1	0.05
*Fringilla coelebs*	1	0.05
*Turdus pilaris*	1	0.05
*Prunella modularis*	1	0.05

**Table 2:** GLMM for the number of great tits at bird-feeders in relation to temperature, habitat (urban or rural), presence of novel object, density of great tits, number of cats, number of dogs, number of pedestrians and the interaction between habitat and presence of object as fixed effects.

Effects	Estimate	SE	*z*	*P*	Effect size
Intercept	0.399	0.324	1.233	0.218	
**Novel object**	**−0.410**	**0.093**	**−4.396**	**<0.0001**	**0.348**
Temperature	−0.017	0.010	−1.769	0.077	0.140
**Habitat: Urban**	**0.315**	**0.091**	**3.466**	**<0.0001**	**0.274**
Early or late trial	0.176	0.102	1.716	0.086	0.136
**Density of great tits**	**0.078**	**0.007**	**10.667**	**<0.0001**	**0.843**
**No. species of birds**	**0.058**	**0.013**	**4.451**	**<0.0001**	**0.352**
**No. cats**	**0.113**	**0.037**	**3.061**	**0.002**	**0.242**
**No. dogs**	**−0.124**	**0.023**	**−5.317**	**<0.0001**	**0.420**
**No. pedestrians**	**0.009**	**0.003**	**3.490**	**0.005**	**0.276**
Habitat * Novel object	**0.535**	**0.119**	**4.485**	**<0.0001**	**0.355**

City was used as a random effect with a variance of 1.16 and a standard deviation of 1.08. The number of observations was 160 and the number of cities 14. Effect size is the z-transformed Pearson product-moment correlation coefficient. Statistically significant terms are shown in bold font.

**Table 3:** GLMM for the total number of birds at bird-feeders in relation to temperature, habitat (urban or rural), presence of a novel object, density of all bird species, number of cats, number of dogs, number of pedestrians and the interaction between habitat and presence of a novel object as fixed effects.

Effects	Estimate	SE	*z*	*P*	Effect size
Intercept	0.978	0.284	3.446	0.0006	
**Novel object**	**−0.270**	**0.075**	**−3.589**	**<0.0001**	**0.284**
Temperature	−0.003	0.008	−0.353	0.724	0.028
**Habitat: Urban**	**0.270**	**0.077**	**3.514**	**0.0004**	**0.278**
Early or late trial	0.107	0.085	1.251	0.211	0.099
**Density of birds**	**0.113**	**0.010**	**11.800**	**<0.0001**	**0.933**
**No. cats**	**0.217**	**0.028**	**7.802**	**<0.0001**	**0.617**
**No. dogs**	**−0.225**	**0.023**	**−9.724**	**<0.0001**	**0.769**
**No. pedestrians**	**0.005**	**0.002**	**1.986**	**0.047**	**0.157**
Habitat * Novel object	**0.276**	**0.099**	**2.783**	**0.005**	**0.220**

City was used as a random effect with a variance of 0.94 and a standard deviation of 0.97. The number of observations was 160 and the number of cities 14. Effect size is the z-transformed Pearson product-moment correlation coefficient. Statistically significant terms are shown in bold font.
